# Microbial genomics challenge Darwin

**DOI:** 10.3389/fcimb.2012.00127

**Published:** 2012-10-12

**Authors:** Didier Raoult, Eugene V. Koonin

**Affiliations:** ^1^Unité de Recherche sur les Maladies Infectieuses et Tropicales Emergentes, Faculté de Médecine, CNRS UMR 6236, IRD 198, Aix Marseille UniversitéMarseille, France; ^2^National Center for Biotechnology Information, National Library of MedicineBethesda, MD, USA

This collection of 14 articles in *Frontiers in Cellular and Infectious Microbiology* aims to re-assess Darwinian and neo-darwinian concepts of biological evolution in the light of the discoveries in comparative genomics of microbes in the twenty-first century. At the time of the publication of the Origins of species in 1859 (Darwin, 1859), Darwin's vision of evolution revolutionized the scientific worldview and even the human perception of the world beyond science. However, a century later, with the consolidation of the Modern Synthesis (neo-darwinism), evolutionary biology has adopted a rather rigid, somewhat dogmatic framework.

Evolutionary biologists have accepted as indisputable truths that the mutations were entirely random. They believe that:

- it was only selection that brought determinism and directionality into the process of evolution
- all evolutionarily consequential heritable changes were extremely small in scale (the principle of gradualism that was staunchly defended by Darwin himself)
- natural selection was the only important factor that shaped the evolving phenotypes and genotypes, driving in particular the emergence of biological complexity
- the history of all life forms, at least in principle, could be adequately described by a single Tree of Life (following the famous sole illustration in the *Origin*) (Dobzhansky, 1937; Huxley, 1942).

At the end of the twentieth and beginning of the twenty-first centuries, genomics, especially comparative genomics of microbes, shattered each of these key tenets of (neo)Darwinism. We are now fully aware that many of the most important genomic changes are by no account miniscule; that the mutational process is far from being completely random; that evolution of complexity via routes distinct from natural selection is possible; and that pervasive horizontal gene transfer makes the original concept of the Tree of Life largely obsolete. Perhaps even more remarkably, the study of genome evolution, in particular in microbes, has brought to fore completely novel aspects of the evolutionary process of which Darwin and the architects of the Modern Synthesis were utterly unaware. Conceivably, the foremost of these phenomena is the unending arms race between cellular life forms and genomic parasites such as viruses and mobile elements that shapes the genomes of both the hosts and the parasites (Raoult, 2010a; Koonin, 2011).

In the nineteenth century, Darwin's concept of evolution was most brutally assaulted by Nietzsche who had a very low opinion of Darwin's work whose rational side, he found, was totally incompatible with life (Nietzsche, 1995). Essentially, Nietzsche believed that Darwin's theories were too simple to be true! Nietzsche's philosophical successors, especially the French postmodern philosophers, extended this radical attack on the dominant concepts of evolution. Among these, Deleuze and Guattari's reflections in “*Le rhizome*” (Deleuze and Guattari, 1976) are inspired by Jacob's work on bacteriophages. Deleuze and Guattari presciently declare that heredity is a mixture of horizontal and vertical descent, and that the origin of all living things was more like a rhizome than a tree. Thus, Deleuze and Guattari seem to have anticipated the key importance of networks in science and life years before the advent emergence of the Internet (Raoult, 2010b). These epistemological insights prepare the ground for acceptance of the revolutionary impact of the twenty-first century discoveries in genomics and epigenomics.

Although the major importance of microbial genomics for understanding evolution is beyond doubt, researchers differ in their opinions as to how radical are the changes brought about by the new discoveries. Some hold that the new discoveries only add details to the neo-Darwinian view of evolution whereas others maintain that the basic tenets of (neo)Darwinism have been falsified in the Popperian sense (Popper, 1959); yet others strive to find a middle ground by positing that, although the bulk of today's evolutionary biology consists of data and concepts that simply did not exist even 30 years ago, at the core there remains the key Darwinian principle of descent with modification.

The present *Frontiers* collection encompasses all these views. In a series of five articles on different aspects of evolution (microbial and beyond) (Georgiades et al., 2011; Colson and Raoult, 2012; Georgiades and Raoult, 2012; Ramulu et al., 2012) including a sweeping overview of modern evolutionary biology (Merhej and Raoult, 2012), Raoult and colleagues promote radical upstaging of the Darwinian paradigm. This proposed overhaul focuses primarily on the Rhizome of Life, the network representation of evolution that under this view is to supplant the Tree of Life. Two articles, by Forterre (Forterre, 2012) and by Gupta and colleagues (Bhandari et al., 2012), present the contrasting, conservative view, that the Darwinian principles remain both necessary and sufficient to understand evolution. Forterre, however, also emphasizes the fundamental importance of cell-virus conflicts that could not have been known by Darwin and his early followers (Forterre, 2012). The review article by Koonin and Wolf (Koonin and Wolf, 2012) strives to balance the radical and the conservative approaches to Darwinian legacy by emphasizing integration more than a paradigm shift *sensu* Kuhn (Kuhn, 1962). Koonin and Wolf outline the fundamental impact of new discoveries while acknowledging Darwinian descent with modification as the surviving core of evolutionary biology. Segerman discusses the major differences in the evolutionary modalities of the stable core and the dynamic compendium of accessory genes in bacteria (Segerman, 2012). Two articles, by Danchin and Rosso (Danchin and Rosso, 2012) and by Aravind et al. (2012) address the effect that gene transfer between eukaryotes and prokaryotes as well as (once again) conflicts between selfish genetic elements and their hosts affect the evolution of eukaryotes. Bertelli and Greub discuss a more specific model system, the phagocytic amoebas and show that these organisms are veritable melting pots of horizontal gene exchange (Bertelli and Greub, 2012).

The Darwinian revolution in the nineteenth century went far beyond the scientific domain and had the broadest philosophical and cultural implications (Raoult, 2010a). It seems appropriate therefore that two articles in the present collection venture outside evolutionary biology and into economic theory and philosophy. Salvucci emphasizes the importance of moving away from simplistic models of market economy, such as those of Smith and Malthus that inspired Darwin to more integrative approaches suitable for analysis of the diverse interactions between different life forms that are central to evolution (Salvucci, 2012). Finally, Baquero and Moya address the problem of intelligibility of complex microbial systems by turning to the ideas of Wittgenstein and advocate the development of complex models that will be commensurate with the complexity of life (Baquero and Moya, 2012).

The philosophical aspect of today's evolutionary biology is especially fascinating. Clearly, we are moving away from the rigid positivist views that dominated rational thinking in Darwin's day and inevitably influenced his thought to a much richer, dynamic philosophical framework of incessant change heavily affected by chance that reverberates with the post-modern thought of the twentieth century but also harks back to the great pre-Socratic philosophers of Greece, Democritus, Parmenides, Heraclites, and Empedocles (Darwin, 1859; Dobzhansky, 1937; Huxley, 1942).

It is our hope that in this collection of articles, the interested reader finds a rich rhizome of ideas that not only summarize the key developments of evolutionary biology in the first decade of the twenty-first century but also might presage some of the directions it will take in the decades to come.
